# The interference of polypharmacy and the importance of clinical pharmacy advice in the treatment of leprosy: a case-control study

**DOI:** 10.1590/0037-8682-0114-2020

**Published:** 2020-06-01

**Authors:** Selma Regina Penha Silva Cerqueira, Lais Sevilha dos Santos, Elaine Faria Morelo, Agenor de Castro Moreira dos Santos, Carlos Augusto Felipe de Sousa, Renata Trindade Gonçalves, Gunter Hans, Daniel da Silva Marques, Raimunda Nonata Ribeiro Sampaio, Patrícia Shu Kurizky, Ciro Martins Gomes

**Affiliations:** 1 Universidade de Brasília, Faculdade de Medicina, Programa de Pós-Graduação em Ciências Médicas, Brasília, DF, Brasil.; 2 Secretaria de Estado de Saúde do Distrito Federal, Laboratório Central de Saúde Pública, Brasília, DF, Brasil.; 3 Universidade de Brasília, Hospital Universitário de Brasília, Brasília, DF, Brasil.; 4 Universidade de Brasília, Faculdade de Medicina, Brasília, DF, Brasil.; 5 Universidade de Brasília, Faculdade de Medicina, Programa de Pós-Graduação em Ciências da Saúde, Brasília, DF, Brasil.; 6 Universidade de Brasília, Núcleo de Medicina Tropical, Programa de Pós-Graduação em Medicina Tropical, Brasília, DF, Brasil.

**Keywords:** Leprosy, Polypharmacy, Therapeutics, Treatment Failure

## Abstract

**INTRODUCTION:**

Although supervised doses are essential for reducing leprosy treatment failure, the impact of specific drug interactions has rarely been assessed. This study aimed to estimate the risk of leprosy treatment suspension in patients receiving polypharmacy.

**METHODS:**

We performed this case-control study in which the primary outcome was defined as the need to discontinue multibacillary leprosy treatment for at least one supervised dose, and the main risk factor was the detection of polypharmacy. Multivariate analysis by logistic regression was used for calculating odds ratio (OR).

**RESULTS:**

This study included 103 patients, of whom 43 needed to discontinue leprosy treatment (hemolysis = 26, hepatitis = 2, hemolysis associated with hepatitis = 6, and suspected treatment resistance = 9) and the rest did not. The severity of drug interactions had no effect on treatment discontinuation. Patients who used five or more drugs in addition to leprosy treatment had almost a 4-fold greater risk of treatment suspension (OR, 3.88; 95% confidence interval: 1.79-9.12; p < 0.001). The number of drugs used also positively influenced the occurrence of hemolysis (p < 0.001). No patient presented evidence of molecular resistance to rifampicin, dapsone, or ofloxacin treatment, as evidenced by genetic sequencing detection of rpoB, folp1, and gyrA mutations.

**CONCLUSIONS:**

Polypharmacy has deleterious effects on the already difficult-to-adhere-to treatment of leprosy and polypharmacy induces hemolysis. Additional measures must be taken to avoid the undesirable effects of inadequate polypharmacy.

## INTRODUCTION

Leprosy is an infectious disease caused by the slow-growing *Mycobacterium leprae* and is manifested by dermatoneurological signs and symptoms such as skin and peripheral nerve damage, which may lead to disabilities of various kinds^1^. The disease is transmitted through direct contact with people having multibacillary leprosy who are not yet under treatment^2^.

The treatment of leprosy should be performed on an outpatient basis, in whatever clinical form, whenever possible, at primary health care facilities^3^. Multidrug therapy (MDT), standardized by the World Health Organization (WHO), comprises a combination of drugs against *M. leprae* and patients are cured after 6 and 12 months of treatment, respectively, for paucibacillary and multibacillary leprosy^4^. Although there is a consensus that supervised doses are essential for reducing the risk of treatment failure, more complex approaches such as polypharmacy are not commonly performed, and the impact of these approaches has rarely been studied^3^.

Polypharmacy is defined as the use of multiple medications to treat various conditions^5^. There is no consensus regarding the exact number of medications in polypharmacy^5^. In the case of leprosy, the high frequency of associated comorbidities^6^ and the need for several drugs to control the sequelae make polypharmacy a relevant concern. Corticosteroids, thalidomide, vitamin D, bisphosphonates, and aspirin are recommended to control leprosy reactions and reduce treatment-related damage^7^. In addition, analgesics, anti-inflammatories, antibiotics, and proton-pump inhibitors are often used in the course of leprosy treatment, introducing an important challenge, especially in primary health care facilities^8^.

Therefore, this study aimed to estimate the risk of MDT suspension in patients subjected to polypharmacy in addition to the treatment of leprosy. We also aimed to describe the frequency and influence of drug interactions during the adequate treatment of leprosy.

## METHODS

We followed the recommendations of the Strengthening the Reporting of Observational Studies in Epidemiology statement: Guidelines for reporting observational studies^9^. From January 2018 to July 2019, all multibacillary leprosy patients treated at the University Hospital of Brasília, Brazil, were recruited. This is a tertiary hospital that is responsible for complex leprosy cases and manages 50% of regional leprosy cases due to the lack of primary care assistance. The criteria for the diagnosis of leprosy conformed to the WHO’s definition^10^.

We performed an unmatched case-control study in which the primary outcome was defined as the presence or absence of the need to discontinue MDT for the treatment of multibacillary leprosy for at least one supervised dose. The main risk factor was defined as the detection of polypharmacy when patients used at least five medications in addition to the drugs used in MDT. This is considered the most commonly used definition of polypharmacy, according to a recent systematic review of the literature^5^. After inclusion, patients were allocated to one of two groups according to their clinical condition as follows: (1) cases: Patients who had to suspend MDT for at least one supervised dose; (2) controls: Patients who did not have to suspend MDT.

Data collection was performed by clinical consultation (medical and nursing consultation). Additional risk factors, such as different levels of polypharmacy, sex, age, degree of disability before treatment, treatment time, adherence to treatment, and occurrence of leprosy reactions, were also reported. The presence of molecular resistance to MDT was accessed by genetic sequencing after nested polymerase chain reaction amplification of rpoB, gyrA, and folp1 sequences. This evaluation has been routinely performed at the hospital for all leprosy patients since 2017, as described elsewhere^11^.

The type of MDT used (standard or substitutive treatment with ofloxacin or/and minocycline) was also analyzed. Drug interaction analysis was performed by a leprosy specialist as well as a pharmacist using the UpToDate Drug Interactions Tool (UpToDate Inc., Wolters Kluwe, Alphen aan den Rijn, The Netherlands) and the Medscape Interaction Checker tool (Medscape, WebMD, New York City, USA). Drug interactions were divided into three types: (1) serious interactions (necessity of drug changes), (2) minor interactions (necessity to monitor possible effects), and (3) no interactions. Intrinsic interactions of the leprosy MDT regimen and interactions between MDT drugs and drugs used to treat reactional states (prednisone and thalidomide) were not considered in this analysis, as those drugs are considered essential for the management of leprosy.

Patients with paucibacillary leprosy, patients belonging to indigenous communities, and patients who did not sign the informed consent form were excluded.

### Sample size

For sample size calculation, we considered previous assistential data from the University Hospital of Brasília. A proportion of patients who previously experienced polypharmacy were chosen. We selected 60% of the cases exposed to polymedication and 30% of controls exposed to polymedication. A bilateral confidence level (1 - alpha) of 95% and power of 80% were also considered. The minimum sample size was 84 patients, 42 in each group. The sample size calculation was performed using the OpenEpi version 3.01 tool (Emory University, Rollins School of Public Health, Atlanta, Georgia, USA).

### Statistical analysis

Numeric variables were not categorized for univariate statistical analysis. Multivariate analysis was performed using logistic regression^12^. The model was constructed using the following variables considered clinically relevant for treatment suspension and medication-induced effects: sex, age, reactional states, presence of serious drug interactions, and the number of drugs used. The threshold for age was set at 60 years to assess the risk of treatment suspension in the elderly population. Odds ratio (OR) were calculated to show the direction and magnitude of the comparisons^12^. To define statistical significance, a 95% confidence interval (95% CI) and a p-value <0.05 were considered. Missing data were ignored at the time of statistical testing, but no patient was excluded for this reason. The RStudio program (RStudio Team [2016]) was used with the EpiTools package - RStudio: Integrated Development for R. RStudio, Inc., Boston, MA URL http://www.rstudio.com/.

### Ethics

Patients were included only after they signed the informed consent form after recruitment. The research complies with the rules established by the Declaration of Helsinki, as well as its revision in 2013^13^. This project was approved by the Research Ethics Committee of the Faculty of Medicine of the University of Brasília (CAAAE: 71029717.1.0000.5558).

## RESULTS

**FIGURE 1:**
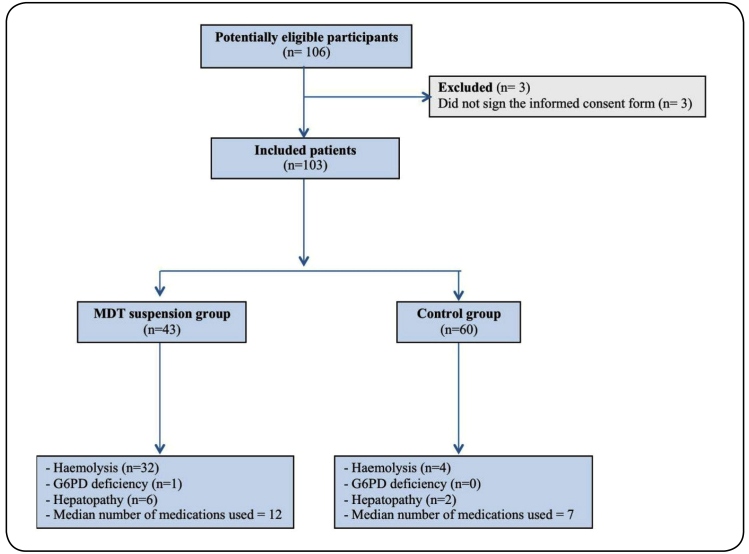
Study diagram of the flow of participants through the study. **MDT:** multidrug therapy for leprosy; **G6PD:** glucose-6-phosphate dehydrogenase.

We included 103 patients in the study. Of these, 43 patients needed to discontinue multidrug therapy (case group), and 60 did not need to discontinue treatment (control group) (Figure 1). The demographic characteristics of each group are presented in Table 1 and Table 2. The proportion of male patients was higher in the control group (p < 0.001) than in the case group. In the univariate analysis, male sex negatively influenced the suspension of MDT (OR, 0.23; 95% CI: 0.10-0.54).

**TABLE 1:** Univariate analysis of possible risk factors for the suspension of leprosy multidrug therapy.

	Leprosy multidrug therapy interruption				
Risk factor	Yes	No	OR	95% CI	p-value
	n/total	n/total			
Male sex	16/43	43/60	0.23	0.10-0.54	<0.001
Substitutive treatment	42/43	8/60	273.00	32.83-2270.42	<0.001
Reactional state	23/43	23/60	1.85	0.84-4.09	0.127
Serious interaction	10/43	7/60	2.29	0.80-6.62	0.118
Minor interaction	41/43	58/60	0.71	0.10-5.23	0.733
Degree of physical disability	--	--	--	--	0.149
Omeprazole use	26/43	30/60	1.53	0.69-3.38	0.395
NSAIDs use	5/43	11/60	0.59	0.19-1.83	0.515
Hydrochlorothiazide use	4/43	4/60	1.44	0.34-6.09	0.717
Metformin use	11/43	4/60	4.81	1.42-16.37	0.010

**n:** number of patients; **OR**: odds ratio; **CI**: confidence interval; **NSAIDs**: nonsteroidal anti-inflammatory drugs.

**TABLE 2:** Influence of numerical variables on the interruption of multidrug therapy (univariate analysis).

	Leprosy multidrug therapy interruption		
Risk Factor	yes	no	p-value
Age: mean (SD)	48.93 (15.86)	45.80 (16.61)	0.339
Consultation number			
Medical: median (IQR)	9 (36.5)	5 (8.25)	0.012
Nursing: median (IQR)	10 (13)	6.5 (9.25)	0.065
Physiotherapy: median (IQR)	2 (4.50)	1 (1.25)	0.019
Bacilloscopic index: mean (SD)	1.26 (1.90)	1.46 (1.92)	0.599
Number of sick contacts: median (IQR)	2 (3)	2 (4)	0.699
Number of medications: median (IQR)	12 (4.5)	7 (4.25)	<0.001

**SD**: standard deviation; **IQR**: interquartile range.

The most common drug-related adverse reactions were hemolysis and hepatopathy. Thirty-two patients from the case group presented with hemolysis, while only four patients from the control group presented with this specific adverse event (OR, 40.73; 95% CI: 11.98-138.51; p < 0.001). The number of drugs used by patients positively influenced the occurrence of hemolysis (p < 0.001). Only one patient in the case group had glucose-6-phosphate dehydrogenase deficiency. Six patients showed signs of hepatopathy, such as transaminase elevation or cholestasis, in the case group, and two patients showed the same reaction in the control group (OR, 4.70; 95% CI: 0.90-24.55; p = 0.065). However, the number of drugs did not influence this reaction (p = 0.540).

The causes of treatment suspension were hemolysis (26 patients), drug-induced hepatitis (two patients), hemolysis associated with drug-induced hepatitis (six patients), and suspected MDT resistance (nine patients). In suspected MDT resistance cases, the treatment was interrupted to perform genetic sequencing after nested polymerase chain reaction sequence amplification of rpoB, gyrA, and folp1 sequences. No patient presented evidence of molecular resistance to rifampicin, dapsone, or ofloxacin treatment.

The drugs most commonly used in addition to MDT were omeprazole, nonsteroidal anti-inflammatory drugs (NSAIDs), hydrochlorothiazide, and metformin, all of which can induce hemolysis (Table 1). In the univariate analysis, only metformin had a positive influence on treatment suspension (OR, 4.81; 95% CI: 1.42-16.37; p = 0.010). However, when adjusted for sex, age, the occurrence of reactional states, serious drug interactions, and the number of drugs used - the same variables used for the main outcome multivariate analysis - metformin use did not show statistical significance for treatment suspension. None of the other most commonly used drugs, in addition to MDT, was found to have an influence on the occurrence of hemolysis in the univariate analysis. An analysis of the influence of other drugs was not feasible because of the multiplicity of factors and low sample variability.

The use of a substitutive regimen with ofloxacin or/and minocycline was strongly associated with the suspension of MDT (OR, 273.00; 95% CI: 32.83-2270.42; p < 0.001) in the univariate analysis. However, this was not considered as a risk factor for the suspension of MDT, as a substitution regimen is a natural choice after adverse reactions and is rarely employed for reasons such as drug resistance. The occurrence of reactionary states, the severity of drug interactions, and the degree of physical disability before treatment did not influence the suspension of MDT (Table 1). The number of medical and physiotherapy appointments was higher in the group that suspended MDT than in the control group (p = 0.012 and p = 0.019, respectively). The number of medications used was also higher in the case group, with a median value of 12 than in the control group, with a median value of 7 (p < 0.001) (Table 2).

The multivariate analysis confirmed that male sex was a protective factor for the suspension of the MDT regimen. Serious drug interactions had no effect on treatment discontinuation. The number of drugs used by the studied patients significantly influenced the chance of MDT discontinuation (Table 3). The patients who took three or more drugs had a 2-fold greater risk of MDT withdrawal (OR, 2.26; 95% CI: 1.42-3.77; p < 0.001). This association became more pronounced as the number of medications increased, where the use of five or more drugs increased the risk of suspension by almost 4-fold (OR, 3.88; 95% CI: 1.79-9.12; p < 0.001), and the use of 10 or more drugs increased the risk of suspension by 15-fold (OR, 15.1; 95% CI: 3.20-83.20). Importantly, these associations were corroborated by considerably narrow confidence intervals (Table 3). In addition, in the multivariate analysis, hemolysis, the most common cause of treatment suspension, was positively influenced by female sex and by the number of drugs used (Table 3).

**TABLE 3:** Multivariate analysis for the assessment of the main risk factor for multidrug therapy interruption in the treatment of multibacillary leprosy.

Outcome	Treatment suspension			Hemolysis			Hepatopathy		
Risk factor	OR	95% CI	p-value	OR	95% CI	p-value	OR	95% CI	p-value
Male sex	0.29	0.11-0.77	0.014	0.20	0.07-0.55	0.002	0.61	0.12-3.17	0.551
Age >60 years	1.20	0.19-7.31	0.843	2.38	0.37-16.40	0.366	1.11	0.07-16.00	0.940
Reactional state	1.05	0.35-3.11	0.936	1.18	0.38-3.72	0.772	2.50	0.43-17.60	0.323
Serious interaction	0.85	0.23-3.08	0.800	1.59	0.45-5.79	0.473	1.56	0.19-9.66	0.646
Number of drugs			<0.001			0.019			0.816
10	15.10	3.20-83.20		6.62	1.39-34.5		0.74	0.05-9.44	
5	3.88	1.79-9.12		2.57	1.18-5.88		0.86	0.22-3.07	
3	2.26	1.42-3.77		1.76	1.10-2.89		0.913	0.41-1.96	

**OR:** odds ratio; **CI:** confidence interval.

## DISCUSSION

Early and adequate treatment of leprosy is the most important measure for controlling disease transmission and managing possible disease sequelae^14^ ^,^ ^15^. Thus, successful administration and completion of all recommended MDT doses are essential. Many factors, including lack of adherence and side effects, frequently disrupt treatment^16^.

The high number of drugs to which a leprosy patient is exposed is a notorious problem. MDT itself is composed of three drugs that are aimed at enhancing the cure probability and avoiding drug resistance^4^. In addition, the treatment of reactional states requires the introduction of additional drugs such as steroids, thalidomide, and pentoxifylline. Furthermore, leprosy treatment creates the necessity for additional drugs such as vitamin D, to prevent deleterious effects of steroids on bone metabolism, and even antithrombotic drugs, pain relievers, and proton-pump inhibitors^7^ ^,^ ^17^ ^-^ ^19^. The problem is compounded if patients present with other comorbidities, such as hypertension and diabetes. We evaluated the effects of polypharmacy on the adequate treatment of leprosy, considering treatment interruption for at least one supervised dose as the main outcome.

In the final multivariate model, we found that male sex was a protective factor against MDT interruption. This can be explained by the fact that men usually have greater body mass than women and that drugs for adults are usually prescribed in fixed doses without considering weight or sex. Patients with a greater body mass than the reference value tolerate some medications better, and this is probably true for polypharmacy^20^. Thus, different metabolic profiles between sexes may be involved in polypharmacy^21^.

The severity of existing drug interactions did not have an effect on the occurrence of treatment interruption. We believe that polypharmacy can lead to serious adverse effects, which are the most common reasons for MDT suspension. The presence of hemolytic anemia was significantly related to the number of drugs used and was ultimately also more prevalent in patients in whom MDT was interrupted than in the control cases. Only one patient showed signs of glucose-6-phosphate dehydrogenase deficiency in the case group. Hemolytic anemia is a well-known drug-related adverse event in the treatment of leprosy because of the use of dapsone, an oxidizing agent^22^ ^,^ ^23^.

Moreover, commonly used drugs such as metformin, acetaminophen, hydrochlorothiazide, ketoconazole, NSAIDs, and omeprazole can cause hemolysis^23^. Possibly, the interactions between two or more of these drugs can enhance hemolytic risk. These drugs have different mechanisms of action, but most are known to produce gastrointestinal side effects. Acetaminophen, an antipyretic, hydrochlorothiazide that inhibits sodium reabsorption in distal renal tubules, and ketoconazole, an ergosterol synthesis inhibitor, can cause hepatotoxicity. NSAIDs, which inhibit prostaglandins, metformin, which reduces glucose absorption, and even omeprazole, a proton-pump inhibitor, can cause unspecific effects, such as nausea.

Regarding limitations, it is important to state that the presently defined treatment suspension is not necessarily related to treatment failure, as a treatment completion delay of 6 months can be tolerated in multibacillary patients^7^. Additionally, a cure is a rather unfeasible targeted outcome for clinical studies because of the long time required for the detection of leprosy recurrence (usually more than 5 years)^24^. However, polypharmacy showed a clear influence on leprosy treatment suspension and the occurrence of hemolysis. Unadjusted confounders in observational studies must also be considered^25^.

We can conclude that polypharmacy has a deleterious effect on the already difficult-to-adhere-to treatment of leprosy. Additional measures, including clinical pharmacist’s advisory functions, must be taken to avoid the undesirable effects of inadequate polypharmacy and to ensure adequate MDT treatment. Further investigations, including prospective studies, must be performed to reduce the undesirable effects of inadequate polypharmacy in the treatment of leprosy patients.

## References

[1] Gurung P, Gomes CM, Vernal S, Leeflang MMG (2019). Diagnostic accuracy of tests for leprosy: a systematic review and meta-analysis. Clin Microbiol Infect.

[2] Job CK, Jayakumar J, Kearney M, Gillis TP (2008). Transmission of leprosy: A study of skin and nasal secretions of household contacts of leprosy patients using PCR. Am J Trop Med Hyg.

[3] de Sousa GS, da Silva RLF, Brasil-Xavier M (2018). Leprosy and primary care: An evaluation study from a medical perspective. Rev Salud Publica.

[4] World Health Organization (WHO) (2018). Regional Office for South-East Asia. Guidelines for the diagnosis, treatment and prevention of leprosy.

[5] Masnoon N, Shakib S, Kalisch-Ellett L, Caughey GE (2017). What is polypharmacy? A systematic review of definitions.. BMC Geriatr.

[6] Butlin CR (2016). The challenge of Multimorbidity in the context of leprosy. Lepr Rev.

[7] Ministério da Saúde (MS) (2016). Diretrizes Para a Vigilância, Atenção e Eliminação Da Hanseníase Como Problema de Saúde Pública: Manual Técnico-Operacional.

[8] do Nascimento RCRM, Álvares J, Guerra AA, Gomes IC, Silveira MR, Costa EA (2017). Polypharmacy: A challenge for the primary health care of the Brazilian Unified Health System. Rev Saude Publica.

[9] von Elm E, Altman DG, Egger M, Pocock SJ, Gotzsche PC, Vandenbroucke JP (2014). The strengthening the reporting of observational studies in epidemiology (STROBE) statement: Guidelines for reporting observational studies. Int J Surg.

[10] World Health Organization (WHO) (2017). Global Leprosy Strategy 2016-2020. Accelerating towards a leprosy-free world. Monitoring and Evaluation Guide.

[11] World Health Organization (WHO) (2017). A guide for surveillance of antimicrobial resistance in leprosy.

[12] Norton EC, Dowd BE, Maciejewski ML (2018). Odds ratios-current best practice and use. JAMA.

[13] Kong H, West S (2013). WMA Declaration of Helsinki- Ethical Principles. World Med Assoc.

[14] Limeira OM, Gomes CM, Morais OOD, Cesetti MV, Alvarez RRA (2013). Active search for leprosy cases in midwestern Brazil: A serological evaluation of asymptomatic household contacts before and after prophylaxis with Bacillus Calmette-Guérin. Rev Inst Med Trop Sao Paulo.

[15] Frade MAC, de Paula NA, Gomes CM, Vernal S, Bernardes F, Lugão HB (2017). Unexpectedly high leprosy seroprevalence detected using a random surveillance strategy in midwestern Brazil: A comparison of ELISA and a rapid diagnostic test. PLoS Negl Trop Dis.

[16] Luna IT, Beserra EP, Alves MDS, Pinheiro PNC (2010). Adesão ao tratamento da Hanseníase: dificuldades inerentes aos portadores. Rev Bras Enferm.

[17] Van Veen NHJ, Lockwood DNJ, Van Brakel WH, Ramirez JR, Richardus JH (2009). Interventions for erythema nodosum leprosum. Cochrane Database Syst Rev.

[18] Van Veen NHJ, Nicholls PG, Smith WCS, Richardus JH (2016). Corticosteroids for treating nerve damage in leprosy. Cochrane Database Syst Rev.

[19] Nagpure S, Kale R, Pathak S, Patel S (2017). A prospective, randomized and double blind once-monthly oral Ibandronate and Risedronate in post-menopausal osteoporosis leprosy patients. IJBAR.

[20] Hanley MJ, Abernethy DR, Greenblatt DJ (2010). Effect of obesity on the pharmacokinetics of drugs in humans. Clin Pharmacokinet.

[21] Chu T (2014). Gender differences in pharmacokinetics. US Pharm.

[22] Deps P, Guerra P, Nasser S, Simon M (2012). Hemolytic anemia in patients receiving daily dapsone for the treatment of leprosy. Lepr Rev.

[23] Rao KV, : DiPiro JT, Talbert RL, Yee GC, Matzke GR, Wells BG, Posey L (2014). eChapter 24. Drug-Induced Hematologic Disorders. Pharmacotherapy: A Pathophysiologic Approach.

[24] Pinheiro MGC, Miranda FAN de, Simpson CA, Carvalho FPB, Ataide CAV, Lira ALBC (2018). Understanding “patient discharge in leprosy”: a concept analysis. Rev Gauch Enferm.

[25] Jager KJ, Zoccali C, MacLeod A, Dekker FW (2008). Confounding: What it is and how to deal with it. Kidney Int.

